# Supplementary material for: Genome-Wide Identification and Characterization of the CC-NBS-LRR Gene Family in Cucumber (Cucumis sativus L.)
Source: Int J Mol Sci. 2022 May 2;23(9):5048. doi: 10.3390/ijms23095048 (PMC9099878; doi:10.3390/ijms23095048)
Supplement: Supplementary file 1 [file ijms-23-05048-s001.zip › Supplementary Files -/Supplementary Figures.pdf]

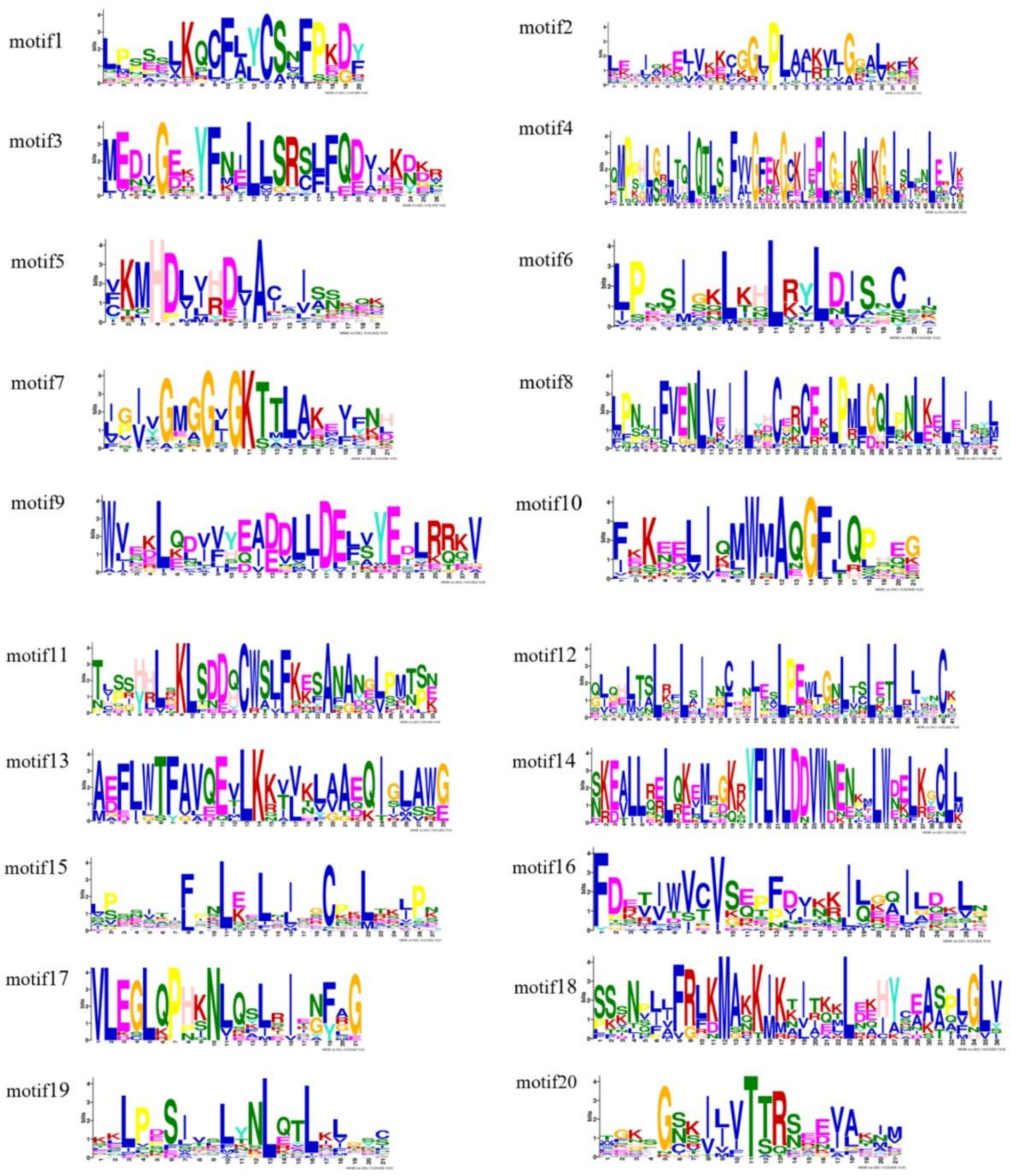

Figure S1 The sequence logos of motifs in CsCC-NBS-LRR.

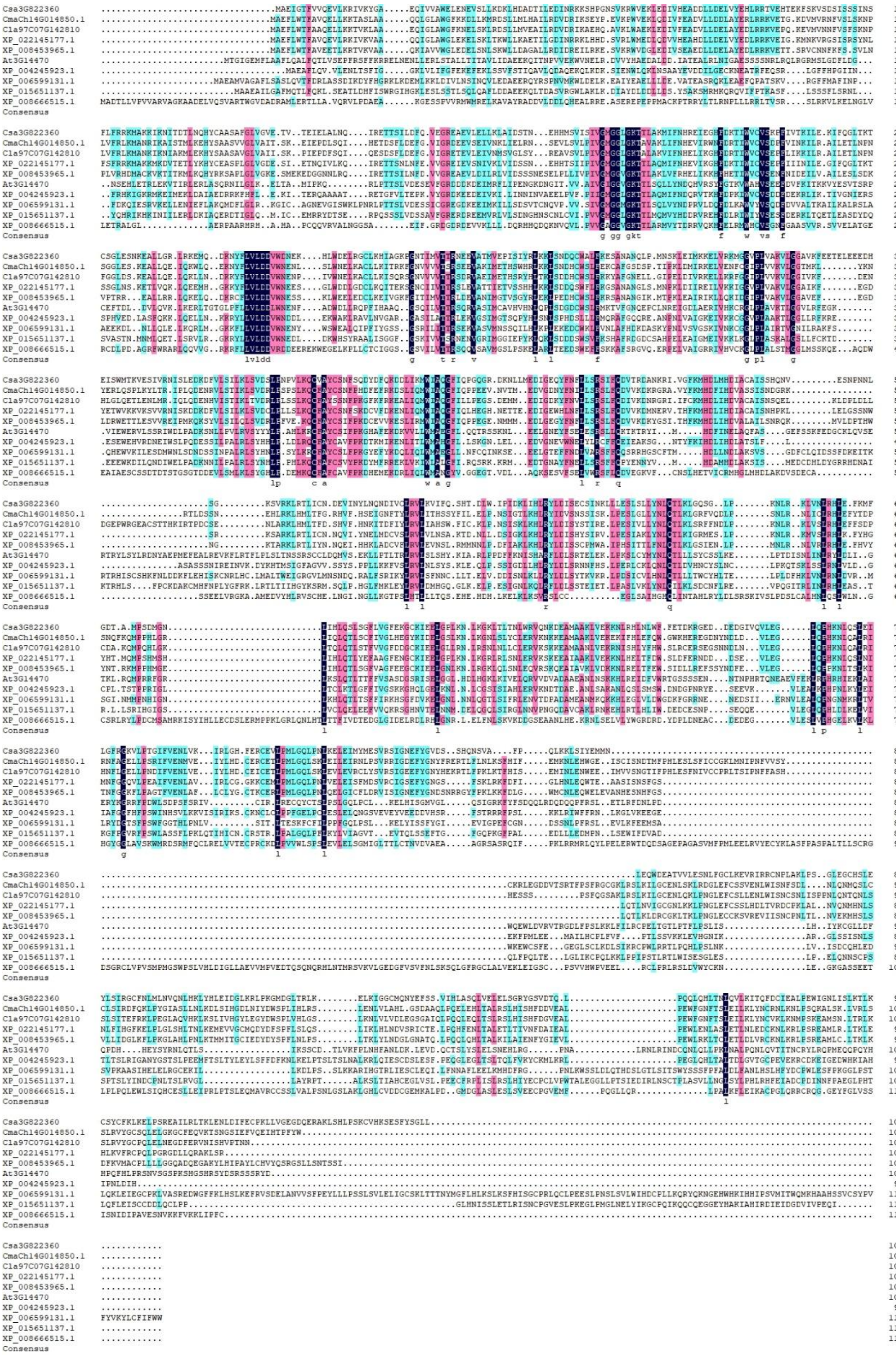

Figure S2 Alignment of the partial CNL proteins between cucumber and other species.

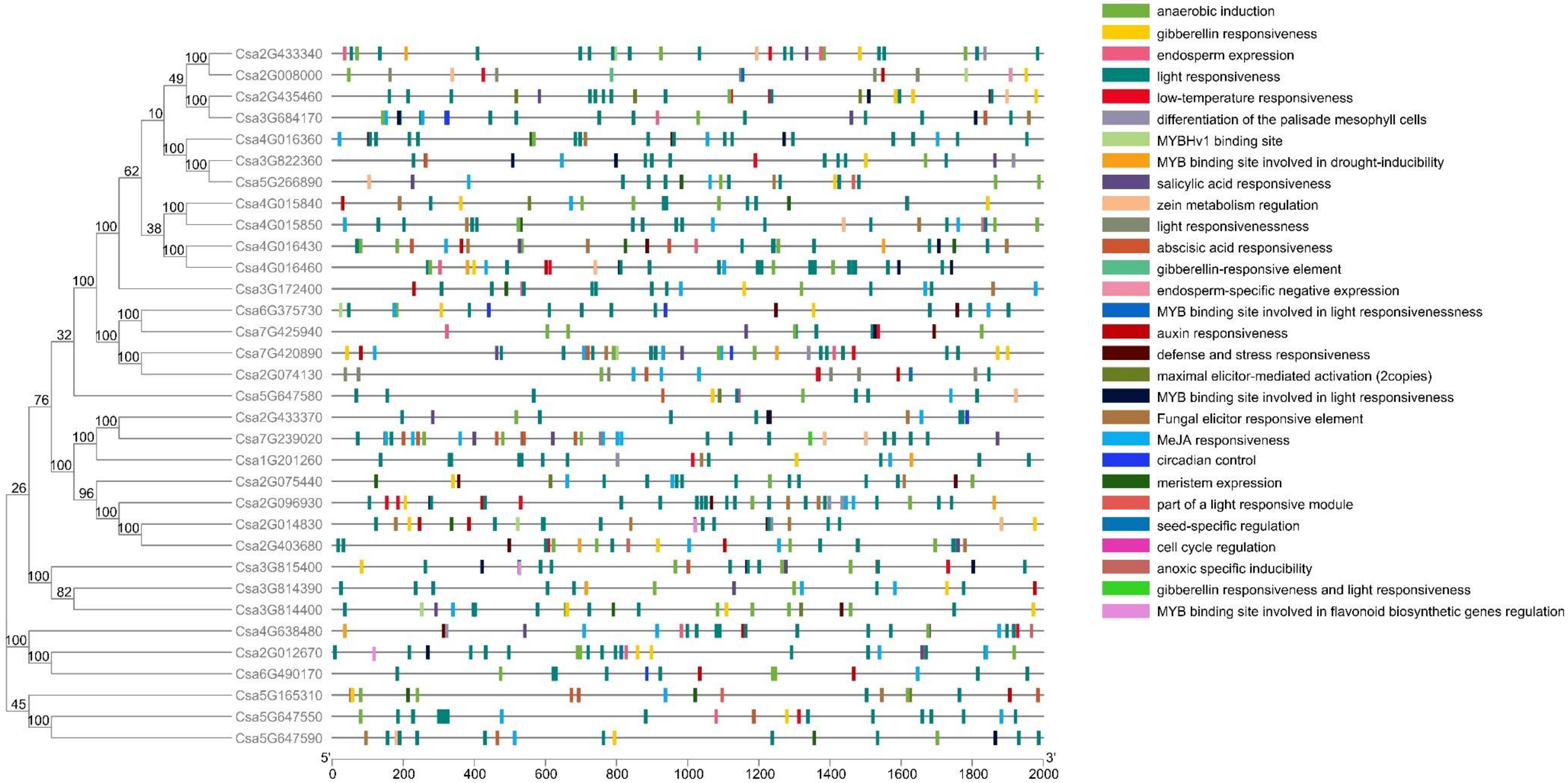

Figure S3 The cis-element of the promoters of the CNL gene in cucumber.
